# Supplementary material for: Outcomes of COVID-19 patients with comorbidities in southwest Nigeria
Source: PLoS One. 2021 Mar 15;16(3):e0248281. doi: 10.1371/journal.pone.0248281 (PMC7959379; doi:10.1371/journal.pone.0248281)
Supplement: Attachment — Submitted filename: Response to Reviewers.docx [file pone.0248281.s003.docx]

January, 2021

Bolajoko O. Olusanya

Academic Editor, PLOS ONE

Dear Dr. Olusanya,

**PONE-D-20-28457 entitled "Outcomes of COVID-19 patients with comorbidities in southwest Nigeria”**

Thank you for your review of our above manuscript and for the comments from the external reviewers. We have carefully revised the manuscript in line with these comments and are pleased to re-submit it for your consideration. In line with your request, we have provided a point-by-point explanation of how we have addressed the comments. We hope these meet your expectation. We look forward to hearing from you.

Sincerely,

Prof Akin Osibogun

For and on behalf of the authors

**JOURNAL REQUIREMENTS**

1. Please ensure that your manuscript meets PLOS ONE's style requirements, including those for file naming.

**Response:** We have ensured that the manuscript from the title page to the references meets the style requirements of the journal.

2. In the ethics statement in the manuscript and in the online submission form, please provide additional information about the patient records used in your retrospective study, including: a) whether all data were fully anonymized before you accessed them and/or whether the IRB or ethics committee waived the requirement for informed consent; b) the date range (month and year) during which patients' medical records were accessed.

**Response:** We have provided the additional information in both the manuscript and the online submission form.

3.We note that you have indicated that data from this study are available upon request. PLOS only allows data to be available upon request if there are legal or ethical restrictions on sharing data publicly… If there are no restrictions, please upload the minimal anonymized data set necessary to replicate your study findings as either Supporting Information files or to a stable, public repository and provide us with the relevant URLs, DOIs, or accession numbers.

**Response:** We have included the minimal anonymized dataset as a Supporting Information file

**REVIEWER 1**

The article is interesting ans is well-written. Conclusions are based on the results and responded to the objective of the study.
Please, specify if you followed the STROBE guidelines for retrospective studies. It is not mentioned in the text.

**Response:** Thank you for your kind comment. We have followed the STROBE statement for reporting observational studies and indicated so in the first sentence under Methods.

**REVIEWER 2**

**Comment #1:**

The manuscript entitled “Outcomes of COVID-19 patients with comorbidities in southwest Nigeria” is an interesting work that adds evidence to the fact that comorbidities are a risk factor for developing complications and evolving to death across different populations, and in this particular case in a cohort of patients in Africa. The manuscript is well written and presents important and interesting results.

**Response:** Thank you for your kind comments.

**Comment #2:**

Methods:
1. Please adhere to the STROBE statement for reporting of observational studies, and state this accordingly in the manuscript.

**Response:** We have followed the STROBE statement for reporting observational studies and indicated so in the first sentence under Methods.

**Comment #3:**

Results:
1. This work is apparently related to this published paper: 10.1016/j.ijid.2020.10.024 . Since some of the results are related I recommend citing them properly.

**Response**: Thank you for picking that. The analyses and write up for this current study and the one cited above were done at the same time, which was why it was not cited originally. However, we have gone ahead to cite it in the methods – reference number 16.

**Comment #4**:

2. Data presented in Figure 1 can be easily summarized in a couple of lines in the text. In order to preserve this Figure, I will recommend making this figure a composite figure with, for example, Panel A showing the frequency of each comorbidity grouped by sex, Panel B the number of comorbidities by age group, and Panel C number of comorbidities and severity at presentation.

**Response:** We have gone ahead to make Figures 1a – 1d which can be combined as a composite Figure 1. The figures depict comorbidities grouped by sex, number of comorbidities by age, by sex and by severity at presentation. Additional report has been included in the results to reflect this.

**Comment #5**:

Discussion:
1. The authors claimed: “This study is the first to determine risks of comorbid conditions among a large sample of COVID-19 patients in Nigeria and one of the first in Africa”. However, other studies have been published in this respect, particularly: PMID: 33296477. I suggest editing this claim accordingly.

**Response:** Thank you for picking that. We have edited the claim to read “This study is presently one of the few to determine risks of comorbid conditions among a large sample of COVID-19 patients in Nigeria and in Africa”

**Comment #6**:

2. The authors claimed that they discovered “that the predominant comorbidities were non-communicable diseases”. However, according to what is stated in the methods, other communicable diseases different than COVID-19 were not assessed in the cohort so making it difficult to sustain this claim. I would suggest to consider this particular issue as a limitation in the study and edit the claim accordingly.

**Response:** We have edited the claim to read “that the predominant comorbidities were hypertension and diabetes”. However, since we assessed communicable diseases namely tuberculosis, HIV and hepatitis B among the cohort, we decided not to consider this as a limitation in the study. Thank you.
